# ‘All those things together made me retire’: qualitative study on early retirement among Dutch employees

**DOI:** 10.1186/1471-2458-13-516

**Published:** 2013-05-28

**Authors:** Kerstin G Reeuwijk, Astrid de Wind, Marjan J Westerman, Jan Fekke Ybema, Allard J van der Beek, Goedele A Geuskens

**Affiliations:** 1 Department of Work, Health & Care, Netherlands Organisation for Applied Scientific Research TNO, Hoofddorp, The Netherlands; 2 Department of Methodology and Statistics, Institute of Health Sciences and the EMGO+ Institute for Health and Care Research, VU University, Amsterdam, The Netherlands; 3 Department of Public and Occupational Health, the EMGO+ Institute for Health and Care Research, VU University Medical Center, Amsterdam, The Netherlands; 4 Body@Work, Research Center on Physical Activity, Work and Health, TNO-VU/VUmc, Amsterdam, The Netherlands

**Keywords:** Early retirement, Pull factors, Push factors, Qualitative study

## Abstract

**Background:**

Due to the aging of the population and subsequent higher pressure on public finances, there is a need for employees in many European countries to extend their working lives. One way in which this can be achieved is by employees refraining from retiring early. Factors predicting early retirement have been identified in quantitative research, but little is known on why and how these factors influence early retirement. The present qualitative study investigated which non-health related factors influence early retirement, and why and how these factors influence early retirement.

**Methods:**

A qualitative study among 30 Dutch employees (60–64 years) who retired early, i.e. before the age of 65, was performed by means of face-to-face interviews. Participants were selected from the cohort Study on Transitions in Employment, Ability and Motivation (STREAM).

**Results:**

For most employees, a combination of factors played a role in the transition from work to early retirement, and the specific factors involved differed between individuals. Participants reported various factors that pushed towards early retirement (‘push factors’), including organizational changes at work, conflicts at work, high work pressure, high physical job demands, and insufficient use of their skills and knowledge by others in the organization. Employees who reported such push factors towards early retirement often felt unable to find another job. Factors attracting towards early retirement (‘pull factors’) included the wish to do other things outside of work, enjoy life, have more flexibility, spend more time with a spouse or grandchildren, and care for others. In addition, the financial opportunity to retire early played an important role. Factors influenced early retirement via changes in the motivation, ability and opportunity to continue working or retire early.

**Conclusion:**

To support the prolongation of working life, it seems important to improve the fit between the physical and psychosocial job characteristics on the one hand, and the abilities and wishes of the employee on the other hand. Alongside improvements in the work environment that enable and motivate employees to prolong their careers, a continuous dialogue between the employer and employee on the (future) person-job fit and tailored interventions might be helpful.

## Background

Similar to other European countries, the average retirement age has increased from 60.9 years in 2001 to 63.1 years in 2011 in the Netherlands [1]. Despite this increase, many workers still retire before the official retirement age of 65 years. Currently, the general population is aging because of decreasing birth rates [2] and increasing longevity [3]. Moreover, the baby boom generation has started to leave work. The increasing ratio of retired persons to the working age population puts pressure on the social security systems in many European countries [4]. For example, in the Netherlands it is estimated that the costs of the General Old Age Pension Act (AOW) will increase from the current 27 billion euros to 47 billion euros in 2040 [5]. Thus, there is a societal need for workers to extend working life.

A transition from work to (non-disability) retirement before the age of 65, i.e. early retirement, can be seen as influenced by so-called push and pull factors [6]. Push factors are defined as negative circumstances that lead to early retirement, such as poor health or lack of job satisfaction [6]. In a recent review of longitudinal studies on determinants of early retirement, poor health and high physical and psychosocial work demands were identified as risk factors for early retirement [7]. These findings from quantitative studies were confirmed in focus groups with employees working in the printing industry [7]. Pull factors are defined as positive factors that attract an individual towards early retirement, such as the desire to spend more time on volunteer work or leisure time activities [6]. In a study conducted among waste collectors and municipal workers, having a partner also increased the likelihood of retiring early [8]. In addition to push and pull factors, the employees’ skills and knowledge may influence the transition to early retirement. Provision of and participation in education and training has been associated with a reduced intention to retire early and actual retirement behavior [9,10]. Moreover, in previous research it has been shown that pension systems offering generous early retirement options encourage early departure from the labor market [11]. Hence, health, work-related factors, skills and knowledge, social factors, and financial factors may influence the transition from work to early retirement.

Despite the current public debate on extending working life, relatively few studies have been performed that explore the factors that influence transitions to early retirement. As a consequence, some push or pull factors may have been overlooked. This is especially true since many of the available studies did not investigate early retirement, but the intention to retire early [7,12,13]. Factors that influence the intention to retire may differ from those that influence actual retirement [14]. In addition, although a variety of factors that predict early retirement have been identified in quantitative studies, little is known on why these factors push or pull individuals to retire early and how they influence the retirement process. This knowledge may contribute to the development of interventions that aim to prolong working life and thus may contribute solutions to the challenges posed by our aging population. Therefore, the present study aimed to explore reasons for retirement before the age of 65 in Dutch employees. Specifically, we investigated which non-health related factors influence early retirement, and why and how these factors influence early retirement.

## Methods

### Design and study population

The present study was part of a larger qualitative investigation on why persons retire early. The role of health in early retirement was extensively described elsewhere [15].

Face-to-face semi-structured interviews with Dutch employees who retired early were conducted. Early retirement referred to retirement before the official retirement age of 65. Persons who left the workforce due to (partially) compensated work disability or unemployment were excluded, since previous research suggests that different factors underlie these transitions out of work [16].

Participants were selected from the Study on Transitions in Employment, Ability and Motivation (STREAM). The aim of this prospective cohort study is to identify under which circumstances persons aged 45 to 64 years prolong their working life, while maintaining good health and good work productivity [17]. Persons were eligible for the present study if (a) they had given permission in the STREAM 2010 questionnaire to be contacted for additional research, (b) had a paid job as an employee at the time of STREAM 2010, (c) had retired before the age of 65 in the last 12 months in 2011, or were going to retire early in the next six months and already formally arranged this with their employer when contacted about the interview, and (d) were aged 58 to 64 years at the time of the interview.

To ensure heterogeneity in the study population, participants were purposefully selected [18] based on age, educational level, and their intention to retire assessed in the STREAM 2010 questionnaire. We selected on age, since different reasons might underlie retirement in those who retired at a relatively young age (e.g. 59 years) compared to those who retired at a higher age (e.g. 64 years). Similarly, educational differences in reasons of early retirement may exist, e.g. due to exposure to different physical and psychosocial working conditions. The intention to retire was assessed with one question in the STREAM 2010 questionnaire, i.e. ‘Are you planning to stop working in the next 12 months?’. This item could be answered on a 5-point Likert scale ranging from ‘certainly not’ to ‘certainly’. Persons who answered ‘maybe’, ‘probably’ or ‘certainly’ were eligible for the present study. We selected purposefully on the intention to retire to assure that both persons in which longstanding processes and persons in which more sudden events influenced early retirement were included.

**Figure 1:**
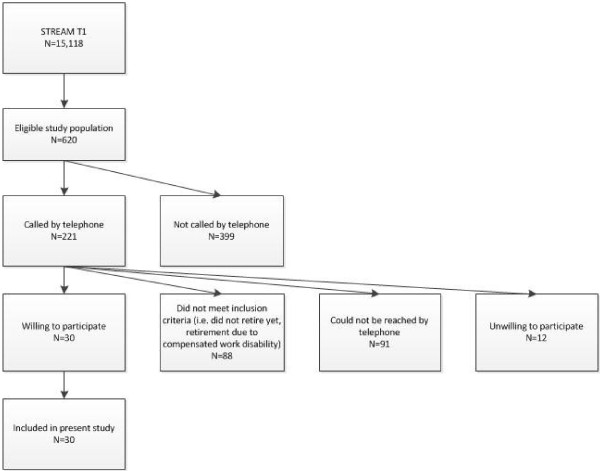
Study population.

In total 620 of the 15,118 persons included in STREAM gave permission to be contacted for additional research, were employed in 2010, and were aged 58 to 64 at the time of the interview (Figure 1). After purposeful sampling on age, education level, and intention to retire in 2010, 221 persons were contacted by telephone between July 2011 and October 2011 to check whether they met the inclusion criteria. The aim and content of the interview study was explained and their willingness to participate in a face-to-face interview was checked. Eighty-eight persons did not meet the selection criteria. They had either not retired yet, or retired early due to compensated work disability. In total 91 persons could not be reached by telephone. These persons were called at least once again after one or two weeks, but could still not be reached. Twelve persons were unwilling to participate. Reasons were personal circumstances (N=4), no time (N=2), unwillingness to talk about work history and early retirement (N=2), and miscellaneous reasons (N=4). Participants were enrolled in the present study by clusters of two to six persons at the same time. In total 30 persons who were eligible and gave permission for an interview were included.

### Interview guide

Prior to the beginning of the study, a comprehensive semi-structured interview guide was created based on the life course perspective [19] and determinants of early retirement according to the literature (Annex 1). The life course perspective considers transitions from work to retirement as a part of the life course. The processes leading to the transition are influenced by someone’s individual history and characteristics, and the context of the transition. The life course perspective has previously been used to understand how persons experience (the transition to) retirement [19]. According to the literature, transitions towards early retirement may be influenced by determinants in the following domains: health, work-related factors, skills and knowledge, social factors, and financial factors [6-11]. The interview guide was tested by means of three role plays of the interviewer with other researchers involved in this study. Subsequently, more examples of in-depth follow-up questions were include in the interview guide.

### Interview procedure

The interviews were carried out by the second author (AdW). The interviewer was familiar with interview techniques, such as clarification, paraphrasing, and summarizing. During most of the interviews, a second interviewer was present who took notes (KR or DR). The interviewers did not have a prior relationship with any of the participants. The interviews were carried out in participants’ homes throughout The Netherlands, except for one person, who, upon request, was interviewed at work. Interviews were digitally recorded. All participants agreed to this procedure.

Before the start of the interview, the interviewer introduced herself, and again explained the aim and content of the interview and subsequent study. Anonymity and confidentiality were assured. Hereafter, open-ended questions were postulated, pertaining to six topics (Annex 1). The first part was aimed at getting acquainted with the interviewee and focused on the personal and home situation. The second part was about the person’s work history and job-job transitions. Together with the participant, the interviewer created a timeline of the interviewee’s work history and other important (positive or negative) events, such as education, marriage, divorce, birth, death of family or friends, and periods of illness. The third part focused on the reasons why an interviewee had retired early, or had made arrangements to do so. Understanding of these reasons was gained through in-depth follow-up questions. The fourth part focused on the timing of the transition from work to early retirement. The fifth part focused on circumstances under which the interviewee would have prolonged his or her working life. The sixth part of the interview concentrated on satisfaction with the transition from work to early retirement. In addition, participants described how they perceived their life in the coming years.

On average interviews lasted 80 min (range: 40–156 min). During 9 interviews non-participants were present (spouse (N=7), spouse and daughter (N=1), and granddaughter (N=1)). In one interview the spouse helped the respondent come up with ideas about what was asked. In two interviews the spouse interfered substantially. Issues brought up by these spouses were interpreted with caution in the analysis.

### Analysis

Analysis of the interviews took place in four steps and in Dutch. First, the interviews were transcribed verbatim. All interviews were listened to at least twice and compared with the transcriptions to check accuracy. Second, 10 interviews were independently summarized using transcriptions and field notes, and were open-coded by AdW and KR. The aim of this step was to understand why and how the transition from work to early retirement had taken place for these persons. Afterwards, AdW and KR discussed summaries, timelines, and codes extensively until consensus was reached about the factors involved in early retirement, and why and how these factors influenced early retirement. If AdW and KR could not reach consensus by comparing their arguments, a third person was consulted and decisive (MW or GG). In the third step, the remaining 20 interviews were summarized, and open-coded by either AdW or KR. Summaries and coded interviews were cross-checked, and AdW and KR regularly met to discuss findings. During these meetings, data saturation was monitored. No new information on reasons of early retirement was derived from the last cluster of five interviews, i.e. from interview 26 to 30. In the fourth step the aim was to investigate how and why the transition to early retirement had taken place in more detail. KR extracted the part in all interview transcriptions in which the transition to early retirement was addressed, and open coded these parts in more detail. These detailed codes were discussed with AdW, and clustered deductively into coding families according to the domains identified in the literature (i.e. health, work-related factors, skills and knowledge, social factors and financial factors) [20]. If codes did not fit into these existing coding families, new coding families were defined, i.e. inductive coding [20].

Parallel to the four steps described above, AdW and KR regularly met to compare interviews on a thematic level. Leading questions during these discussions were: (a) what similarities can be identified between interviewees’ experiences?, and (b) why did certain processes take place in some persons, but not in others? To enhance robustness of the findings, main results were also discussed with other project members (MW and GG). In order to manage the data of the interviews, the computer package for qualitative analysis Atlas.ti 6.1.17 [21] was used.

### Ethical considerations

The Medical Ethics Committee of the VU University Medical Center Amsterdam declared that no ethical approval was needed to conduct this study. Informed consent was obtained verbally from all participants during the telephone conversation in which persons were invited for the interview.

## Results

**Figure 2:**
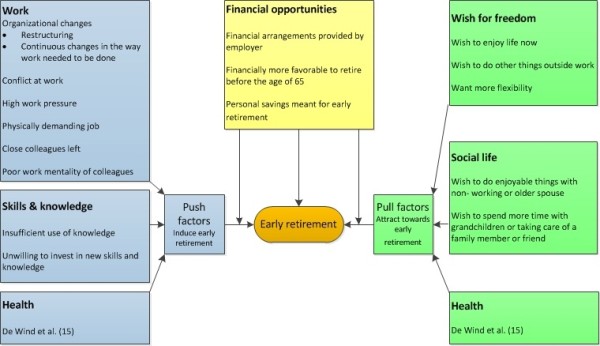
Factors involved in early retirement.

Characteristics of the study population are reported in Table 1. The participants’ jobs before retirement varied and included both blue and white collar jobs, such as mechanic, manager, and teacher. Different reasons for early retirement were reported, namely factors that pushed employees out of work to early retirement, factors that pulled employees towards early retirement, and financial factors. An overview of these factors is presented in Figure 2. In most persons an interplay of factors played a role.

**Table 1:** Characteristics of the study population

**Characteristic**		**Total sample (N=30)**
Women	N (%)	6 (20%)
Age (years)	Median (range)	62 (60–64)
Retirement age (years)	Median (range)	61 (60–64)
Education level		
Low	N (%)	12 (40%)
Moderate	N (%)	4 (13%)
High	N (%)	14 (47%)

N = number of participants.

### Factors that pushed towards early retirement

#### Work

Work-related factors were frequently described as a reason for early retirement (Figure 2). Changes in the work organization, e.g. restructuring, often preceded early retirement. One man (64), who worked in the welfare sector, reported that after recurrent restructuring, the department he worked for closed down. He felt that due to his age, he would be unable to find a new job. He had been rejected one year earlier for another position (*“But yeah, then they want some young person”).* In his view, due to this lack of opportunities, retirement was unavoidable. When he was asked why he had retired before the age of 65, he answered:

“Well, there was no perspective anymore. As of July 1st the department I worked in was closed.”

Another man (61), who worked as a mechanic, reported that when enterprise restructuring occurred, his employer played a substantial role in his early retirement process. His employer decided not to fire him, but to offer an early retirement arrangement:

“Last year I was almost fired due to a reorganization but then administration said […] as of next year he can already retire early, and receive his pension, firing him will take a few months anyways so we might as well just keep him. Well that’s what they did then.”

In addition to large organizational changes, continuous changes in the way work needed to be done was reported as a reason for early retirement. Employees became tired of continuous changes in work tasks and the need for these changes was not always fully understood. This decreased their motivation to continue working.

Conflicts at work were mentioned as a reason to leave the workforce before the age of 65 as well. A woman (64), who had an administrative job, described that she did not enjoy her work as much as she had before when a conflict with her colleagues arose. She explained:

“Couldn’t get on that well with my colleagues. Or they couldn’t get on with me […] sometimes it clashed […] that was very unpleasant, no, not nice at all.”

Furthermore, high work pressure and physically demanding work were reported as push factors for early retirement, because they reduced the ability to continue working until an older age in a particular job. A technician (60), who worked offshore in engine rooms of freighters and oil rigs around the world, explained that the physical burden of his job did not allow him to continue working until the age of 65. Although he was given the opportunity to get an office job as a clerk, he was not willing to perform this type of work. He was offered a favorable financial arrangement by his employer and retired early. He argued:

“When I was around 40 I already noticed […] the first of the wear and tear. And then you think, guys, I won’t make it to 65.”

#### Skills and knowledge

Factors related to skills and knowledge were mentioned as push factors towards early retirement. Some employees were not willing to invest in their careers anymore, and, for example, retired early before they had to take a new course or training. Others described that they were dissatisfied with the limited use of their skills and knowledge, which decreased their motivation to work for the company, and pushed them out of the workforce. A man (64) who worked in the welfare sector argued that he had a lot of knowledge that, he felt, was not sufficiently used by his supervisors:

“I had a lot more knowledge than others […], so from their point of view […] I would have said: make use of that. I was actually ‘used’ way too little […], and that of course also gives a certain negative feeling […], and then you give up earlier.”

### Factors that pulled towards early retirement

#### Desire for freedom

Many respondents described that they wished to do other things outside of work, wanted to enjoy life, or looked forward to more flexibility in their life. As these wishes gained importance, respondents became less motivated to continue working and were more attracted to early retirement. A woman (60) who worked as a physical therapist explained:

“And also enjoying yourself. I think that with physical therapy work you are really inflexible, […] for people with office jobs […] with nice weather they can say I’m taking the afternoon off, going to the beach, well we couldn’t do that because you were fully booked and the following week as well. So I think it’s really restricting.”

A 62 year old economics teacher said:

“I think that after working for 40 years it’s now time for other things. And aside from that I wanted something else, I wanted to be more flexible with my time.”

#### Importance of family and friends

Respondents mentioned that as they got older, spending time with family or friends became more important to them. An older or non-working spouse often pulled the employee towards early retirement, since respondents wished to spend more time together. A woman (60), who worked in the health care sector her whole career, emphasized:

“The fact that (my partner) is 10 years older than I am is decisive for my stopping work at an earlier age […], if I want to do fun things, then I shouldn’t keep working until I’m 65.”

Some persons were attracted to early retirement because they wished to take care of a partner, family member (e.g. grandchild), or friend. A primary school teacher (female, 61) described:

“And by now I have grandchildren. That is also one of the reasons that I stopped a little sooner, because I’m going to be babysitting soon.”

### Financial factors

In addition to the ‘push’ and ‘pull’ factors towards early retirement described above, financial factors influenced early retirement (Figure 2). Most employees had the opportunity to opt for early retirement schemes (e.g. financial arrangements provided by the employer or sector, flexible early retirement schemes), which made early retirement accessible. Others saved money to facilitate an early exit from working life. In some persons, financial opportunities to retire early became important in the context of other push and pull factors, whereas financial opportunities played a more direct role for others. In all cases, the financial opportunity to retire early was essential in the final decision to leave the workforce before the age of 65. Some described that they had known for years at what age they would qualify for early retirement arrangements and changed their mindset accordingly. A man (60), who worked in the police force, explained:

“I knew during my contract time that I could retire at 60 […] then it turned into 62, but if you participated in the life-course savings scheme you could do it early.”

A troubleshooter in machine construction (61) who enjoyed his job, described his financial opportunity to retire as follows:

“The Social Benefit taxes that I paid, that bag of money was laying there. I can use that and if I don’t, then at 65 it’s gone […] that money that I saved […] all those years, someone else will use it. And then I say, no that is my money, I’m using it.”

## Discussion

For most employees, a combination of factors played a role in the process towards early retirement, although the specific factors involved differed between individuals. Push factors towards early retirement included, among others, organizational changes at work, conflicts at work, high work pressure, high physical job demands, and dissatisfaction with the limited use of one’s particular skills and knowledge. Pull factors towards early retirement included the wish to do other things outside of work, enjoy life, have more flexibility, spend more time with a spouse or grandchildren, and care for others. In addition, the financial opportunity to retire early played an important role for all respondents.

Our findings on the influence of work-related factors, the wish to do other things outside of work, and financial factors are in line with previous qualitative and quantitative studies on early retirement [7,12]. The present study also identified an additional factor, namely insufficient use of older workers’ skills and knowledge. Moreover, the present study provided new insights into how and why different factors influenced early retirement. Push factors towards early retirement seemed to cause early retirement via a decrease in motivation, ability, and opportunity to continue working. For example, insufficient use of skills and knowledge decreased a person’s motivation to continue working and physically demanding work reduced an employee’s ability to continue working until the age of 65. Our results suggest that employees who felt unable to find a new job due to their age when confronted with a push factor experienced a reduced opportunity to continue working, and as a consequence, retired early. In line with this, earlier studies have shown that age discrimination impacts the opportunity for older workers to remain in or re-enter the workforce [22]. Pull factors towards early retirement, such as spending more time with a significant other, mainly influenced early retirement via an increased motivation to retire early. Moreover, financial factors, such as favorable retirement schemes, importantly influenced the opportunity to retire before the age of 65.

In line with previous research [23], the process towards early retirement appeared to be multi-factorial and was frequently not determined by one single factor. This suggests that interventions and policies should not focus on one factor but integrate measures on a combination of relevant factors. When ranking the relative importance of factors involved in the early retirement process, financial factors appeared to be most important and were often a precondition for early retirement. Push and pull factors seemed of equal importance for early retirement in our study population. We recommend that future quantitative research investigates the relative importance of factors involved in early retirement in different groups of workers to shed more light on the potential of interventions.

Since different factors played a role for different persons, it seems that especially interventions tailored to the individual and the specific working conditions may support the prolongation of working life. Work-related interventions can address both push and pull factors, though the intervention potential may differ between these factors. Push factors towards early retirement can be targeted directly, whereas pull factors relate to private life, and hence, can only be accommodated. With respect to push factors, work-related interventions could include measures that improve working conditions such as work pressure, social climate and use of individual’s knowledge. The impact of organizational and task-related changes on early retirement stresses the importance of a working environment that supports maintaining a high employability and flexibility throughout employees’ careers. With respect to pull factors, work-related interventions are recommended to include measures that match working conditions with factors pulling individuals towards early retirement [24,25]. Flexible working hours could for example fit with the wish to spend more time with a spouse or take care of others and maintain a satisfactory work-life balance. To ensure a good fit between the demands of the job and the ability and wishes of the employee, a dialogue between employers and employees may be helpful from an early phase in the career onwards.

Due to the aging of the population and changes in retirement scheme regulations, early retirement schemes will become financially less favorable in the Netherlands in the near future. As a consequence, the opportunities to leave the workforce early will decrease. Most participants in this study still had the opportunity to opt for favorable retirement schemes, and it would be interesting in future research to explore whether reasons for early retirement will shift when these arrangements become less accessible. It could be hypothesized that push factors towards early retirement will gain importance relative to pull factors such as the wish to do other things outside of work. However, in the present study, employees who could financially afford to retire early in order to focus on other aspects of life experienced this as a positive outcome. Besides, it could be hypothesized that some employees may leave the work force via different pathways in the future, e.g. unemployment. These potential consequences further necessitate improvements in the working environment including flexible working arrangements, policies supporting employability (e.g. skills), and improvements in labor market opportunities for older persons. Another area that could be further researched is on how to balance the societal need to prolong working life due to the aging of the population and the older worker’s preferred work-life balance. This is especially important because satisfaction with the job relates to health and well-being [26]. Future research on early retirement also needs to take the employers perspective into account; employers may, for example, be confronted with costs associated with the loss of older skilled workers and recruitment of new workers, but also with costs associated with retention of older workers.

The qualitative character of the present study was considered a strength, since it allowed us to gain insight into how various factors led to early retirement. This method also allowed respondents to report important factors that were not yet identified in the literature and played a role in their retirement process. Another strength of the current study was the study population, since only employees who had retired early, or already formally arranged with their employer to do so within six months, were included. Hence, actual early retirement was explored rather than the intention to retire early.

The present study also has some limitations. Firstly, in qualitative studies the researcher is an important instrument in data collection and analysis [27], which may have influenced the findings. Therefore, analysis of the interviews was predominantly done by two persons. Moreover, to ensure robustness of findings, members of the project team discussed data quality and results. Secondly, during the interviews, persons looked back at their transition from work to early retirement. There is a risk of recall bias and transformation of the ‘real’ story, since persons may not remember facts correctly or may be influenced by psychological processes, such as cognitive dissonance. The interviewer used in-depth follow-up questions to validate interviewees’ answers. Moreover, to prevent biased results we checked for inconsistencies in the stories and interpreted these parts with caution. Thirdly, during some interviews a spouse, daughter, or granddaughter was present. This may have influenced the participants’ answers. To limit bias, issues brought up by non-participants were interpreted with caution in the analysis. Fourthly, in the present study, differences between subgroups, e.g. gender and educational level, could not be investigated. Fifthly, it should be acknowledged that country specific pension systems may influence both the accessibility and factors involved in early retirement. This might limit the generalizability of (some of) our findings to other (non-European) countries. Finally, before retirement, some persons had expected to miss pleasant aspects of working life including social contacts with colleagues, day rhythm, and appreciation (i.e. pull factors towards work). This made the decision to retire early tough. However, we were unable to identify reasons to *continue* working life, since we did not include employees who stayed in the workforce until the age of 65 years in the present study.

## Conclusion

In conclusion, this study found that the process towards early retirement is multi-factorial. Apart from financial incentives, the prolongation of working life may be supported by improving the fit between the physical and psychosocial job characteristics on the one hand and the abilities and wishes of employees on the other hand. Work-related interventions that enable and motivate employees to prolong their careers may include measures that reduce physical and psychosocial load, support employees in coping with organizational changes and maintain employability, support the use of older workers’ skills and knowledge, and offer the opportunity to perform activities outside of work (e.g. flexible working hours). Tailored interventions seem especially important, since a different combination of factors resulted in early retirement for different persons. Therefore, a continuous dialogue between employers and employees on the (future) person-job fit and tailored interventions might be helpful in promoting prolonged working lives for older employees.

## Annex

### Annex 1 - Interview guide

#### I. Background information

1. To get to know you a little bit better, I would like you to tell me something about yourself…

•What is your home situation like?

•What kind of family do you come from?

#### II. Work history

2. Could you describe what types of jobs you have had in the past?

Draw, with the interviewee, his/her career history on a timeline.

If respondent mentions different jobs:

3. What was the reason for the job change?

•Why?

•Since when?

•What triggered this?

•How did this happen?

4. For what reasons did you work in this job for x years?

If respondent mentions one job

5. For what reasons did you work in this job for x years?

6. Did you work part-time or fulltime?

7. What is your educational background? Do you have any diplomas?

8. Could you mention something about your home situation during your career?

9. Did you participate in any activities outside of work, such as hobbies, volunteer work, or taking care of others?

•How important were those activities to you?

10. How important was work to you?

11. Did this change throughout the years?

•Why?

•Since when?

12. Could you describe your most recent job?

#### III. Reasons for retiring early

13. What were reasons for you to retire early?

If someone mentions (changes in) health (examples):

What kind of health problems did you have?

•Since when?

•For what reason didn’t you continue working with the health problems?

If someone mentions (changes in) work stress or work-related tasks (examples):

Why did your work become more stressful?

•Since when?

•Why was that?

How did you experience it when teams were combined?

•Why was that?

How did you experience it when you got a new team leader?

•Why was that?

If someone mentions (changes in) how their skills and knowledge matched with the job demands (examples):

How did you experience it when you could not keep up with new developments?

•Since when?

•Why was that?

If someone mentions (changes in) the social situation (examples):

How did you experience it when your partner stopped working?

•Why was that?

How did you experience it when you became a grandmother/grandfather?

•Why was that?

If someone mentions (changes in) financial situation (examples):

How did you experience it that you were financially able to stop working sooner?

•Why was that?

•How were you able to?

How did you experience it that you were offered an appealing financial incentive from your employer?

•Why was that?

If someone mentions (changes in) the ability to work (examples):

Why were you no longer able to work?

•Since when?

•Why weren’t you able to work anymore since then?

If someone mentions (changes in) their motivation to work (examples):

Why did your motivation to work change?

Why did your work become less / more important to you?

•Since when?

•Why did your motivation to work change at that moment?

Why were you no longer satisfied at work?

•Since when?

•Why were you no longer satisfied at work at that moment?

If someone mentions (changes in) their opportunity to work (examples):

Why was there no longer an opportunity for you to work?

•Since when?

•Why did you perceive a decrease in the opportunity to continue working?

14. You mentioned several reasons why you retired early.

The interviewer summarizes these reasons.

Is this correct?

Are there any other important reasons for which you retired early?

15. What were the most important reasons for you to retire early?

16. From the literature we know that aside from …, and …, other factors can also play a role in early retirement decisions.

Fill in blanks on the basis of the interview.

If health was not mentioned:

For example, health. If you look back, did health play a role in your early retirement ?

If work-related factors were not mentioned:

For example, work-related factors. If you look back, did work-related factors play a role in your early retirement?

If skills and knowledge were not mentioned:

For example, skills and knowledge. If you look back, did skills and knowledge play a role in your early retirement?

If social factors were not mentioned:

For example, social factors. If you look back, did social factors play a role in your early retirement?

If financial factors were not mentioned:

For example, financial factors. If you look back, did financial factors play a role in your early retirement?

If ability was not mentioned:

For example, sometimes people are no longer able to work. If you look back, did this play a role in your early retirement?

If motivation was not mentioned:

For example, sometimes people are no longer motivated to continue working, or they no longer want to continue working. If you look back, did this play a role in your early retirement?

If opportunity was not mentioned:

For example, sometimes people no longer had the opportunity to continue working. If you look back, did this play a role in your early retirement?

#### IV. Timing of the transition

If someone retired early within the past 12 months:

17. On xx-xx-xxxx you retired early. Why did you retire at that moment specifically?

•Why not sooner?

•Why not later?

18. Why did the factors that you mentioned before lead to your retiring at that moment?

OR You mentioned that x, x, and x played a role in your retiring early. Did something change in those factors that led you to retire on xx-xx-xxxx?

19. After you decided to retire early, you still worked x months. What kind of expectations did you have of those last x months?

20. To what extent were those expectations of the last × months accurate?

If someone will retire early within the next 6 months:

21. On xx-xx-xxxx you are going to retire early. Why are you retiring on that moment specifically?

•Why not sooner?

•Why not later?

22. When did you and your employer discuss your retiring early?

23. Could you explain how that went?

•Who initiated the process?

•Why was the process initiated?

•How long did this take?

24. Why did you then decide that you would keep working x months?

•Why not longer?

•Why not shorter?

#### V. Circumstances under which one would have continued working

25. If you could have decided yourself, would you have retired early on xx-xx-xxxx, or would you have worked longer or shorter?

•Why?

26. Did it feel like it was your own decision to retire early?

•Why?

27. Are there any circumstances under which you would have continued working? Under which circumstances would you have continued working?

If someone mentions circumstances:

28. Why would you have worked longer under those circumstances?

If someone doesn’t mention circumstances

29. Why did you not want to / could you not continue working?

If someone mentions multiple circumstances (question 3):

30. You mentioned various circumstances under which you would have continued working. *The interviewer summarizes these.* Is that correct?

•Which circumstances were most important?

#### VI. Current situation and future

If someone retired early within the past 12 months:

31. You have now been retired for x months. How do you feel about it?

32. What were your expectations about early retirement?

33. To what extent does your early retirement compare to these expectations?

34. Now I have another very open question to conclude with. How do you feel about the future?

•Do you see this as a positive thing?

•Do you have plans for the future?

If someone will retire early within the next 6 months:

35 In x months you will retire early. What are your expectations about these upcoming months?

36. Soon you will retire early. What are your expectations about early retirement?

37. Now I have another very open question to conclude with. How do you feel about the future?

•Do you see this as a positive thing?

•Do you have plans for the future?

## Competing interests

The authors declare that they have no competing interests.

## Authors’ contributions

All authors made a substantial contribution in the design of the present study. KR and AdW were responsible for data collection. KR and AdW analyzed the interviews and KR, AdW, GG, and MW regularly discussed the findings. All authors read and approved the final manuscript.

## Pre-publication history

The pre-publication history for this paper can be accessed here:

http://www.biomedcentral.com/1471-2458/13/516/prepub
